# Patient and caregiver perspectives on quality of life in dementia: Evidence from a South Asian population

**DOI:** 10.1371/journal.pone.0285701

**Published:** 2023-05-18

**Authors:** Surangi Jayakody, Carukshi Arambepola

**Affiliations:** 1 Division of Health Sciences, Warwick Medical School, University of Warwick, Coventry, United Kingdom; 2 Department of Community Medicine, Faculty of Medicine, University of Colombo, Colombo, Sri Lanka; KPC Medical College and Hospital, INDIA

## Abstract

Dementia has become a public health priority along with population ageing worldwide. Owing to its chronic progressive nature in the absence of a cure, maintaining the best possible quality of life (QOL) has become the desired outcome for people with dementia. The aim of this study was to compare the Quality of Life (QOL) of patients with dementia in Sri Lanka when assessed based on the patient’s and caregiver’s perspectives. A cross-sectional study was conducted among 272 pairs of patients with dementia and their primary caregivers recruited systematically from the psychiatry outpatient clinics of tertiary care state hospitals in the district of Colombo, Sri Lanka. The QOL was assessed using the 28-item DEMQOL among patients and the 31-item DEMQOL-proxy among primary caregivers. The total QOL ratings and subscale scores obtained by patients and caregivers were compared and assessed for the significance of the mean scores using the independent t-test and of the mean difference in ratings using the Wilcoxon test. Agreement between patients and their caregivers on the ratings for QOL was also assessed using the Bland Altman plot. The mean overall QOL score according to patient ratings (mean = 79.7; SD = 12.0) was significantly higher than the caregiver ratings (mean = 70.6; SD = 12.3) (p< 0.001). Mean scores for the four subscales (positive emotion, negative emotion, memory, and daily life) were also significantly higher according to the patient’s ratings (p<0.001). Total scores obtained by patients and their caregivers showed a positive and significant correlation (r = 0.385; p<0.001). Bland Altman plot demonstrated acceptable agreement between their ratings. The study confirms the ability of dementia patients with mild to moderate severity to successfully rate their own QOL. Furthermore, the caregiver’s ratings cannot be substituted for the patient’s ratings and vice versa.

## Introduction

Population aging has become a universal phenomenon. It adds ‘years to life’ but not necessarily ‘life to years’, as reflected by the devastating impact of age-related diseases such as dementia, that are commonly seen among the elderly [1]. In dementia, effective treatment is successful only in reducing the disease progression marginally. However, improving the quality of life (QOL) has shown to minimize dementia-related morbidities to a great extent [2–4]. Hence, there is growing consensus to focus more on providing care to dementia patients to improve their QOL, and therefore to assess QOL as a crucial health-related outcome in dementia [3, 5, 6].

Sri Lanka is a country in South Asia, with impressive health indicators achieved despite its low GDP. It has a well-established health care system that provides extensive curative and preventive services free of charge, yet this system is not well-geared to handle medical and social issues of the rapidly expanding elderly population in the country, notably that of dementia patients [7]. As with other countries in South Asia, dementia has many implications in Sri Lanka, requiring urgent attention. In finding solutions, the first step would be to identify the magnitude of the issue, by assessing the current status of the QOL of patients with dementia, which could in turn provide strong evidence for policymakers to advocate effective measures for improving the care in dementia.

Studies conducted on dementia-specific QOL are sparse in South Asia apart from a study on determinants of QOL among dementia patients [8]. Although there have been many studies from developed countries [2–4], this evidence cannot be directly applied to developing countries owing to the differences in the care provided. For example, unlike in developed countries where care for dementia patients is provided mainly through well-established hospital- and community-based care systems, South Asian countries depend mainly on informal family-based care provided through non-paid caregivers, which could in turn affect dementia-specific QOL [8].

Another major concern in assessing the QOL in dementia wherever in the world has been the poor reliability of self-reported information obtained from patients. Consequently, most QOL assessment tools tend to rely on proxy reports rather than on patients’ views. However, since QOL is best measured from a patient’s perspective [9], such ratings may predominantly reflect the caregivers’ perspective rather than of the patients, thus leading to misinterpretation of the actual QOL [10–13]. Therefore, especially in today’s context where greater emphasis is placed on ‘patient-centered care’, patients’ own perceptions of the QOL are also expected to play an equally important role at least in mild to moderate dementia [14]. This highlights the need to focus on population-specific differences in the QOL assessed both by patient and caregiver in relation to dementia. As per the definition of QOL by the World Health Organization, which is ‘an individual’s perception of their position in life in the context of the culture and value systems in which they live and in relation to their goals, expectations, standards, and concerns, the cultural aspects, values and spirituality also seem to add to the complexity of QOL measurements [1]. In this background, the aim of this study was to describe and compare the QOL of patients with dementia in Sri Lanka when assessed based on the patient’s and caregivers’ perspectives.

## Material and methods

A cross-sectional study was conducted in the premier tertiary care state hospitals in the district of Colombo, Sri Lanka. These hospitals, which represent the most well-equipped health facilities in the country, including specialists in neurology and psychiatry whose expertise is sought for providing care for patients with dementia, were selected in order to achieve the highest patient yield. The study population consisted of pairs of patients with dementia accompanied by their primary caregivers, who were being followed up in outpatient psychiatry clinics during the last six months. Patients had their diagnosis made according to the DSM-IV classification (confirmed by documental evidence such as diagnosis card and clinic records). Those with severe dementia, institutionalized patients (e.g. elderly homes), patients with speech or hearing impairment, or any other diagnosed psychiatric condition which could compromise their ability to participate in an interview were excluded. To exclude those with severe dementia, the Mini-Mental State Examination (MMSE) [15] was performed on each eligible person by a consultant psychiatrist and confirmed by an MMSE score of less than 10. A primary caregiver was defined as the person who had been taking primary responsibility for a patient and being with him/her at least during the preceding six months.

The minimum sample required was calculated [16], in order to detect an expected proportion of patients with ‘good’ QOL of 0.2 based on the clinical experience of specialists in psychiatry who provide care for patients with dementia, in the absence of literature for Sri Lanka; 1.96 of 95% confidence level; and precision of 0.05. The sample was increased to 280 to account for 15% non-response. The eligible and consenting pairs of patient-caregiver were recruited systematically from the neurology and psychiatry outpatient clinics until the desired sample size was obtained.

The QOL was assessed using the 28-item DEMQOL among patients and the 31-item DEMQOL-proxy among primary caregivers. Responses of each item were on a four-point Likert scale [from 1 (a lot) to 4 (not at all)], giving a total score range of 28–112 for DEMQOL and 31–124 for DEMQOL-proxy. Higher scores indicated better QOL. Both these tools had been translated to local languages, culturally adopted, and validated previously in a similar setting. Both instruments have demonstrated an acceptable level of judgmental and construct validity. Reliability of both DEMQOL (Cronbach’s alpha = 0.87; r = 0.864) and DEMQOL-proxy (Cronbach’s alpha = 0.874; r = 0.834) was also well-established [17].

The study was approved by Ethics Review Committee, Faculty of Medicine, University of Colombo, Sri Lanka (Reference no: EC-16-200). Informed written consent form all the participants were obtained prior to data collection.

### Data analysis

Data were analyzed using Statistical Package for Social Science (SPSS) Version 20. Total QOL ratings and subscale scores of the patients and caregivers were compared using the following methods: mean and standard deviation (SD) assessed for significance using an independent t-test and Wilcoxon test to assess the significance of the mean difference in ratings; Spearman correlation coefficients (r) to describe the correlation between the two ratings; and Bland Altman plots to assess the agreement between patients and their caregivers on the ratings given for QOL, in which the difference between the two ratings (Y-axis) was plotted against the mean of the same two ratings (X-axis) [18]. Proportional bias, which occurs ‘when one measure gives values that are higher (or lower) than those from the other by an amount proportional to the level of measured variable’ could distort the agreement between the two rating scales. Linear regression analysis was performed to assess this bias [19].

## Results

The overall response rate was 97.1% (N = 272). There were 164 patients with mild dementia (60.3%) and 108 with moderate dementia (39.7%). Their mean age was 72.6 years (SD = 7.05). As shown in Table 1, most of the patients were females (57.4%), educated below Grade 10 (58.4%), and of middle social class (76.8%). Caregivers consisted of the patients’ children (44.1%), spouses (35.3%), and siblings (15.4%). The majority of them were females (67.3%), below 55 years of age (46%) and educated beyond the General Certificate of Education (GCE) Ordinary Level (59.9%).

**Table 1:** Socio-demographic characteristics of the pairs of patients with dementia and their primary caregivers (N = 272).

Socio-demographic characteristics	Dementia patient		Caregiver	
	No.	%	No.	%
**Age (in years)**				
≤ 55	0	0.0	125	46
56–65	43	15.8	58	21.3
66–75	148	54.4	88	32.4
≥ 76–80	81	29.8	1	0.3
**Sex**				
Male	116	42.6	89	32.7
Female	156	57.4	183	67.3
**Ethnicity**				
Sinhalese	246	90.4	246	90.4
Tamil& Moor	26	9.6	26	9.6
**Current marital status**				
Married	145	53.3	240	88.2
Single	39	14.3	23	8.5
Widowed	88	32.4	9	3.0
**Highest level of education**				
Grade 1–5	65	23.8	14	5.1
Grade 6–10	94	34.5	77	28.3
Passed G.C.E Ordinary Level	66	24.3	67	24.6
Passed G.C.E Advanced Level	36	13.2	96	35.2
Degree or Diploma	11	4.0	18	6.6
**Monthly family income (in Rupees)**				
No permanent income	41	15.1	12	4.4
10,000 or less	48	17.6	17	6.2
10,001–20,000	95	34.9	98	36.0
20,001–30,000	80	29.4	112	41.1
More than 30,000	8	2.9	33	12.1
**Social class**				
Low	61	22.4	61	22.4
Middle	209	76.8	209	76.8
High	2	0.7	2	0.7

### Comparison of the QOL scores between patients and their caregivers

The mean overall QOL score according to the patient ratings (mean = 79.67; SD = 12.04) was significantly higher than the caregiver ratings (mean = 70.57; SD = 12.28) (p< 0.001) (S1 Table).

When comparing the pairs using Wilcoxon Signed Rank Test, 207 patients had a significantly higher total QOL score than their caregivers, while only 60 caregivers showed higher scores than their patients (Table 2).

**Table 2:** Mean ranks of DEMQOL and DEMQOL-proxy total scores according to Wilcoxon Signed Rank Test (N = 272).

Ranks	No.	Mean rank	Sum of ranks	Significance
Negative ranks (QOL pt.<QOL-proxy)	60	98.3	5897.5	Z = 9.496 p<0.001
Positive ranks (QOL pt. >QOL proxy)	207	144.4	29880.5	
Ties (QOL pt. = QOL proxy)	5			

### Agreement between the QOL ratings among patients and their caregivers

**Fig 1:**
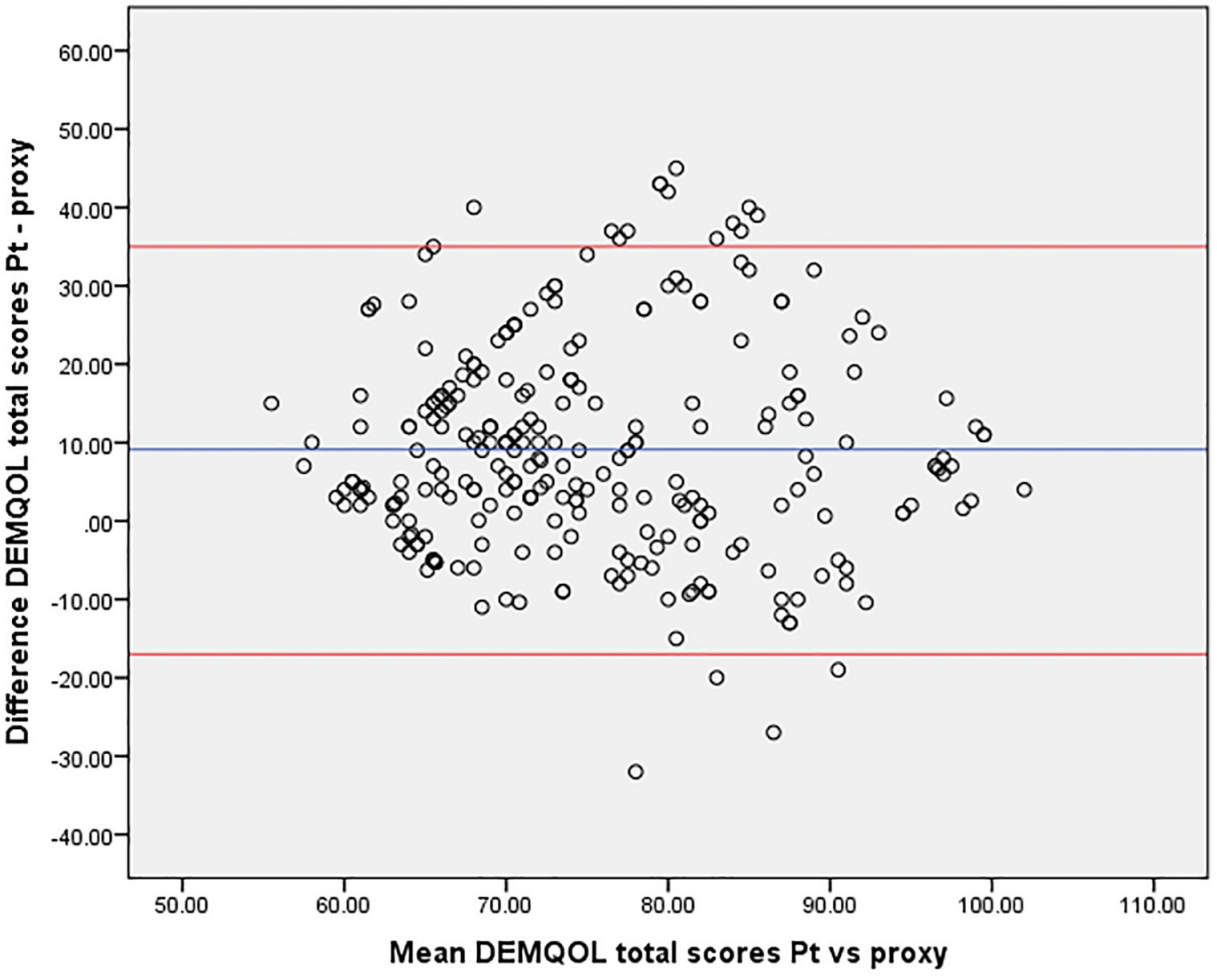
Bland Altman plot between patient and caregiver mean QOL scores.

Total scores obtained by patients and their caregivers further showed a positive and significant correlation (r = 0.385; p<0.001), as shown in the Bland Altman plots (Fig 1) which indicate the mean difference between patients and caregivers (blue line) and 95% confidence limits (red lines). The plot demonstrated acceptable agreement between the ratings made by patients and caregivers.

Further, linear regression of the QOL scores revealed a non-significant p-value (p = 0.71), hence accepting the null hypothesis (mean difference does not equal zero), suggesting no proportional bias between the DEMQOL and DEMQOL-proxy ratings made by the patients and their caregivers (S2 Table).

When individual scores obtained for the four subscales (positive emotion, negative emotion, memory, and daily life) were compared (Table 3), patients showed significantly higher scores compared to their caregivers except for the ‘memory subscale’.

**Table 3:** Comparison of the QOL scores of individual subscales between patients and caregivers.

Subscale		Mean score	SD	t value	Significance (2-tailed)	Mean difference	95% CI of the difference
Positive emotion	Pt.	11.85	3.14	9.02	< 0.001	2.34	1.83–2.86
	CG	9.50	2.92				
Negative emotion	Pt.	22.59	4.64	21.0	< 0.001	7.87	7.13–8.61
	CG	14.72	4.07				
Memory	Pt.	16.32	3.39	-9.3	< 0.001	-3.45	-4.18–-2.72
	CG	19.78	5.10				
Daily life	Pt.	27.50	4.30	20.87	<0.001	7.30	6.61–7.99
	CG	20.19	3.84				

Pt.–Patient with dementia CG- Caregiver

## Discussion

The current study assessed the QOL of patients with dementia and their caregivers attending tertiary care state hospital clinics in the district of Colombo, Sri Lanka. To our knowledge, this is the first study done in South Asia on this aspect, while considering both the patients’ and caregivers’ perspectives of QOL.

The QOL related to dementia in Sri Lanka, as assessed by the patients themselves was shown to vary on a wide scale (95% CI; 61, 105), with a mean score of 79.7 (SD = 12.0). For comparison, there was no other study available from South Asia. In comparison with developed countries, the mean QOL score reported based on the original DEMQOL in a sample of patients with dementia in the UK was 91.22 (SD = 11.11) within a similar scatter (range = 63–108) [1]. This difference in QOL scores is likely to be due to the socio-economic and service-related differences in the two countries. On one hand, in South Asian countries like Sri Lanka, only a few dementia-care facilities are available; and these too are fee levying so that the majority cannot access the services they require. Also, on the other, caring for the elderly is considered a strong traditional custom in Asian countries, with social taboos attached to care-homes, so that the dementia patients predominantly live with their own families despite the socio-economic constraints the families face in caring for a patient with dementia.

With regards to the caregiver perspective on QOL, the mean score obtained was 70.56 (SD = 12.28), which was a poorer rating than that of patients with dementia (p<0.001). Observing this type of significant difference in scores between the patient and caregiver is in line with some previous studies [10, 13]. Higher patient-reported mean QOL scores were reported compared to proxy-reported scores using QOL-AD in a study done in Norway (mean difference = 5.2; p<0.001) [4] and in another done in Switzerland among patients with Alzheimer’s disease (mean difference = 4.3; p<0.001) [20]. These differences could be a reflection of the different perspectives the patients and care providers would have on the QOL in dementia [21]. This phenomenon is seen not only in dementia, but also in other chronic diseases such as stroke, and has been discussed in the context of “disability paradox”–‘a tendency for people with persistent disability to report good or excellent QOL even if their caregivers are not in agreement’ [22]. This point highlights the need for considering the patients’ perceptions as well, when providing care to improve dementia specific QOL.

The UK study where the original study on QOL was conducted based on DEMQOL also reported lower QOL scores of caregivers than that rated by the patients, but with no considerable difference [1]. It should be noted that unlike in other studies, patients for this original study had been recruited from a wide range of care arrangements such as dementia residential care-home facilities, day care-home facilities and nursing homes [1]. Such availability of care-plans is shown to drastically reduce the caregiver burden, which in turn has shown to improve the ratings made by caregivers on patients’ QOL [23]. Furthermore, family-caregivers’ assessment on the QOL as opposed to that of non-family caregivers could be a direct reflection of the status of these patients prior to illness. In this backdrop, the largest care-related gap in dementia is not the care per-se, but lack of social support. This is a crucial healthcare provision that requires attention in South Asia.

Although there is a positive correlation in the current study between the QOL scores of patients and caregivers, it does not necessarily imply the agreement between the two ratings. However, the Bland Altman plots indicate that most of the differences between the patient and caregiver in total QOL scores are dispersed within the 95% confidence limits, indicating acceptable agreement [18]. A study conducted in Australia has shown similar results using Bland Altman plots. Patients and caregivers showed good agreement (scatter lying within 95% confidence limits) in their QOL ratings according to QOL-AD, despite the total scores of patients being higher than those of caregivers [10]. This implies that patient’s perspectives on QOL are informative especially when providing person-centred care, thus proxy reports cannot substitute for patient reports, except in severe dementia [10]. Also, these findings suggest that it is rational to use the ratings of patients, and use the ratings of caregivers only as complementary, in mild to moderate dementia.

### Limitations

Since this study was based on patients being followed up at psychiatry clinics in Colombo District, it does not reflect the QOL of the under-diagnosed or under-treated patients living in the community who are likely to have poorer QOL. Hence, the results cannot be generalized to all dementia patients in the country. Nevertheless, considering the rarity of the disease in the community and the clinic patients having a definitive diagnosis made, the hospital setting appears to be most appropriate for this study. Further, adhering to strict eligibility criteria as well as using a locally validated and reliable tool to assess the QOL in dementia have maximized the internal validity.

## Conclusions and recommendations

When applied to patient-caregiver dyads, DEMQOL and DEMQOL-proxy demonstrate the potential both as a simple and low-cost tools that could be used to assess the current level of QOL in patients with dementia in clinics as well as in field settings. Study findings further confirm the ability of dementia patients with mild to moderate severity to successfully rate their own QOL. In addition, it concludes that the caregiver’s ratings cannot be substituted for the patient’s ratings and vice versa.

## Supporting information

S1 Table Descriptive statistics of the DEMQOL and DEMQOL-proxy overall scores. (DOCX)

S2 Table Results of linear regression analysis to assess the proportional bias. (DOCX)
