# Supplementary material for: Patient and caregiver perspectives on quality of life in dementia: Evidence from a South Asian population
Source: PLoS One. 2023 May 18;18(5):e0285701. doi: 10.1371/journal.pone.0285701 (PMC10194915; doi:10.1371/journal.pone.0285701)
Supplement: S1 Table — (DOCX) [file pone.0285701.s001.docx]

***S1 Table:* *Descriptive statistics of the DEMQOL and DEMQOL-proxy overall scores***

| **Scale** | **Minimum** | **Maximum** | **Median** | **Mean** | **SD** | **95% CI** |
| --- | --- | --- | --- | --- | --- | --- |
| DEMQOL | 61 | 105 | 77.3 | 79.7 | 12.0 | 78.3-81.1 |
| DEMQOL-proxy | 48 | 100 | 68.0 | 70.6 | 12.3 | 69.1-72.0 |
