# Supplementary material for: Patient and caregiver perspectives on quality of life in dementia: Evidence from a South Asian population
Source: PLoS One. 2023 May 18;18(5):e0285701. doi: 10.1371/journal.pone.0285701 (PMC10194915; doi:10.1371/journal.pone.0285701)
Supplement: S2 Table — (DOCX) [file pone.0285701.s002.docx]

***S2 Table: Results of Linear Regression Analysis to Assess the Proportional Bias***

| **Model** | **Unstandardized Coefficients** | | **Standardized coefficients** | **t** | **Significance** |
| --- | --- | --- | --- | --- | --- |
|  | **B** | **SE** | **Beta** |  |  |
| (Constant) | 11.382 | 6.146 |  | 1.852 | 0.065 |
| Mean difference | -0.030 | 0.081 | -0.023 | -0.374 | 0.709 |

Dependent variable: difference score
